# The Current Achievements of Multi-Gene Panel Tests in Clinical Settings for Patients with Non-Small-Cell Lung Cancer

**DOI:** 10.3390/cancers16091670

**Published:** 2024-04-25

**Authors:** Tadashi Sakaguchi, Akemi Iketani, Seiya Esumi, Maki Esumi, Yuta Suzuki, Kentaro Ito, Kentaro Fujiwara, Yoichi Nishii, Koji Katsuta, Hiroki Yasui, Osamu Taguchi, Osamu Hataji

**Affiliations:** 1 Department of Respiratory Medicine, Matsusaka Municipal Hospital, 1550, Tonomachi, Matsusaka 515-0073, Mie, Japan; 2 Pathology Department, Matsusaka Municipal Hospital, 1550, Tonomachi, Matsusaka 515-0073, Mie, Japan

**Keywords:** Amoy Dx^®^ Pan Lung Cancer PCR panel, multi-gene panel test, next-generation sequencing, non-small-cell lung cancer, Oncomine Dx Target Test

## Abstract

**Simple Summary:**

Performing multi-gene testing for detecting driver oncogene alterations is essential to avoid missing treatment opportunities with appropriate targeted therapies, including in cases with rare driver oncogene alterations. However, multi-gene testing has not been sufficiently implemented under the condition that the Oncomine Dx Target Test is only available in clinical settings in Japan. This study evaluated the recent status of multi-gene panel tests in our institution under the condition that both the Oncomine Dx Target Test and Amoy Dx^®^ Pan Lung Cancer PCR panel were available. A favorable submission rate and success rate of multi-gene testing were shown, along with a favorable detection rate in recent clinical settings. As our practice of multi-gene testing has matured and the number of options for multi-gene testing with different characteristics has increased, it has become feasible to perform multi-gene testing on the majority of patients with non-small-cell lung cancer.

**Abstract:**

Some multi-gene panel tests have been implemented in clinical settings to guide targeted therapy in non-small-cell lung cancer (NSCLC) in Japan. The current performance of multi-gene panel tests under the condition that the Oncomine Dx Target Test (ODxTT) and Amoy Dx^®^ Pan Lung Cancer PCR panel (AmoyDx-multi) are available remains relatively unknown. We retrospectively reviewed consecutive patients with NSCLC, whose FFPE samples were considered for genetic testing. We assessed the submission rates, the success rates, and the driver oncogene detection rates of multi-gene panel tests. A total of 225 patients were histologically newly diagnosed with NSCLC or diagnosed with a recurrence of NSCLC without a previous multi-gene panel test at our institution. Among the 225 patients, the FFPE samples of 212 patients (94.2%) were submitted for multi-gene panel testing, including 191 samples (84.9%) for the ODxTT and 21 samples (9.3%) for the AmoyDx-multi. Among the 212 samples submitted to multi-gene panel tests, the success rate was 99.5% (211/212). The detection rate of driver oncogene alterations for all histologies was 52.4% (111/212), and that for adenocarcinoma was 69.7% (106/152). A favorable submission rate and success rate of multi-gene panel tests were shown, along with a favorable detection rate in recent clinical settings.

## 1. Introduction

The identification of targetable driver alterations and the development and clinical application of targeted therapies for non-small-cell lung cancer (NSCLC) are progressing rapidly. For NSCLC harboring driver alterations, targeted therapy markedly improves patient outcomes and quality of life [1,2]. The appropriate screening of driver alterations has therefore become a necessary step for patients diagnosed with NSCLC.

As the number of driver oncogene alterations recommended for detection in clinical settings has increased, it has become difficult to analyze all of the recommended genes using multiple single-gene tests due to increased tissue consumption and increased genetic testing costs [3]. Furthermore, the success rates of the ordered tests have decreased [4]. A recent report suggests that a multi-gene panel test performed prior to the initiation of systemic treatment can potentially enhance prognosis by detecting a wider range of driver oncogene alterations than multiple single-gene tests [5]. The importance of performing a multi-gene panel test for comprehensive biomarker analysis has therefore increasingly been relied upon to avoid missing treatment opportunities with appropriate targeted therapies, including in cases with rare driver oncogene alterations.

The Oncomine Dx Target Test (ODxTT) (Ion Torrent PGM Dx Sequencer; Thermo Fisher Scientific, Waltham, MA, USA) is one of the NGS panels that can detect a total of 46 genes, including 35 genes by DNA sequence and 21 genes by RNA sequence simultaneously. It has been approved by the Ministry of Health, Labor and Welfare of Japan since February 2019 as a companion diagnostic for targeted therapies on four driver alterations: EGFR mutations, ALK fusions, ROS1 fusions, and BRAF V600E mutations [6]. In addition, RET fusions were added as a companion diagnostic of the ODxTT in clinical use in September 2021 in Japan, and the use of the ODxTT for the detection of HER2 mutations was additionally approved in May 2023. Following clinical use of the ODxTT, the Amoy Dx^®^ Pan Lung Cancer PCR panel (AmoyDx-multi) (Amoy Diagnostics Co., Ltd., Xiamen, China), a multi-PCR panel which can detect a total of 11 genes, including 4 genes using DNA and 7 genes using RNA, has been approved for use in Japan since June 2021 as a companion diagnostic for targeted therapies on four driver alterations: EGFR mutations, ALK fusions, ROS1 fusions, and BRAF V600E mutations. Additionally, MET Ex.14 skipping mutations, KRAS G12C mutations, and RET fusions have been approved as companion diagnostics of the AmoyDx-multi since August 2022, November 2022 and March 2023, respectively. Each of these multi-gene panel tests has its own characteristics, including the number of genes to be tested, the range of detectable variants, the limits of detection (LOD) for each gene, and the turnaround time (TAT), defined as the period from submission for the genetic tests to the result reporting. Each of these multi-gene panel tests also has its own appropriate sample criteria to be submitted, including the amount of nucleic acid required, quality controls, and recommended tumor content. It is recommended that FFPE specimens be submitted for both tests, as this is required to assess tumor content.

In general, the samples submitted for multi-gene panel tests are required to be of more sufficient quantity and better nucleic acid quality than for conventional single-gene tests to avoid unsuccessful analysis [7,8,9]. The analysis of multi-gene panel tests using inappropriate specimens may lead to erroneous test results and lost treatment opportunities for patients harboring driver alterations, resulting in significant disadvantages for the patient’s prognosis. To ensure promising results from the use of multi-gene panel tests, more stringent sample handling is required than for conventional single-gene tests. Although the success rates and submission rate of the ODxTT have gradually increased due to efforts aimed at obtaining sufficient tumor samples and appropriate sample handling in each institution [10,11], few reports mention the current achievements of multi-gene panel tests, including the submission rates, success rates, and detection rates of driver oncogene alterations, under the condition that AmoyDx-multi is also available.

In this study, we retrospectively evaluated the recent status of multi-gene panel tests in our institution under the condition that both ODxTT and AmoyDx-multi were available.

## 2. Materials and Methods

### 2.1. Patient Selection

This retrospective study was conducted at Matsusaka Municipal Hospital, Japan. We reviewed electronic data from consecutive patients who were either histologically newly diagnosed with NSCLC or were histologically diagnosed with a recurrence of NSCLC without previous multi-gene panel test analysis in our institution from March 2022 to December 2022. Referred patients whose samples obtained at other hospitals had been submitted for genetic tests and patients diagnosed only with cytological samples were excluded. Clinical data assessments included patient characteristics, clinical stage, sampling methods, pathological findings, and the results of genetic tests. This study was performed in accordance with the Declaration of Helsinki. This study was approved by the institutional review board of Matsusaka Municipal Hospital (IRB number J-238-230420-3-2) on the 22 April 2023. Informed consent was obtained through an opt-out method.

### 2.2. Sampling Methods and FFPE Sample Preparation

Small tissue samples collected by endobronchial biopsy/transbronchial biopsy (EBB/TBB), transbronchial needle aspiration (TBNA), fine-needle aspiration (FNA), computed tomography-guided percutaneous needle biopsy (CTNB), and pleural biopsy were immediately placed in 10% neutral buffered formalin (NBF) and fixed for about 12 to 24 h at room temperature (RT). In samples obtained by TBNA and FNA, core tissues were handled as tissue samples and fixed with formalin as well [12]. In cases of surgical resection, we took 10 mm × 10 mm samples in tumor-rich areas for genetic testing concurrently when sampling for intraoperative rapid diagnosis (IRD), and the samples were immediately placed in 10% NBF and fixed over 24 to 48 h at RT for appropriate formalin fixation [13]. If sampling for genetic testing was difficult due to a small tumor volume in cases of limited resection surgery, including lung segmentectomy and wedge resection, all the limited resection samples were injected with 10% NBF using a syringe and immediately put in 10% NBF after sampling for IRD and fixed as above. In cases where the surgical resection was performed on a Friday, the duration of formalin fixation was permitted for up to 72 h because large samples obtained by surgical resection are prone to RNA analysis failure due to inadequate formalin fixation [11,12]. Formalin-fixed tissues underwent serial processing and were embedded in paraffin to create formalin-fixed and paraffin-embedded (FFPE) blocks with meticulous care to avoid nuclease contamination. For small tissue samples, a couple of samples were embedded in an FFPE block. The number of tumor cells and the tumor content of the samples stained with hematoxylin and eosin were evaluated by skilled cytopathologists. Macro-dissection and marking were performed as needed.

Regarding genetic test selection in our institution, samples were submitted for the ODxTT if the tumor content was ≥30% after marking and macro-dissection as needed, which was the recommended tumor content for testing, the number of tumor cells was more than 200 cells in a section, and the presence of necrosis was less than 20% [14]. If the tumor content was between 20% and 30% after marking and macro-dissection, the number of tumor cells in a section was between 100 and 200 cells, the presence of necrosis was between 20% and 50%, or the prompt results of the genetic tests were required in a case of clinical emergency, the samples were submitted for AmoyDx-multi testing. Samples which failed to meet the above conditions due to low tumor content, an insufficient number of tumor cells, or excessive necrosis were considered unsuitable for multi-gene testing and were either submitted for feasible single-gene testing only or re-biopsy was considered. Regarding the number of sections for submission to multi-gene testing, 5 to 10 slide-mounted 8 µm sections of the surgical samples and 10 to 20 slide-mounted 8 µm sections of small biopsy samples, depending on tumor amount, as shown in Table 1, were submitted to LSI Medience Laboratories (Tokyo, Japan). LSI Medience Laboratories performed the ODxTT based on Thermo Fisher’s Ion AmpliSeq technology and the AmoyDx-multi tests based on real-time PCR in accordance with the manufacturer’s instructions. In cases of clinical emergencies, in-house AmoyDx-multi testing was performed in accordance with the manufacturer’s instructions because its TAT was shorter than the outsourced inspection of the AmoyDx-multi and ODxTT.

### 2.3. Outcomes

We evaluated the submission rates, success rates, and detection rates of the driver oncogenes of the tests. The analysis results were regarded as successful if all of the results of the companion diagnostics for each test were completely available: EGFR, ALK, ROS1, BRAF, and RET for ODxTT and EGFR, ALK, ROS1, BRAF, and MET Ex14 skipping for AmoyDx-multi. Analysis results were regarded as unsuccessful if the sample did not pass the nucleic acid concentration threshold, if one or more of the companion diagnostic results mentioned above were invalid due to a failure to meet the DNA and RNA sample quality control (QC) metrics or “No Call” results for the ODxTT, or if the sample did not pass any of the thresholds of internal controls for RNA and external controls for DNA for the AmoyDx-multi test. The driver genes for the detection rate were evaluated using the genes detectable for both the ODxTT and AmoyDx-multi, including genes other than the companion diagnostics: EGFR, ALK, ROS1, BRAF, RET, MET Ex14 skipping, KRAS, NTRK, and ERBB2.

## 3. Results

### 3.1. Sample Characteristics

A total of 229 patients were newly diagnosed with NSCLC or diagnosed with a recurrence of NSCLC without a previous submission of a multi-gene panel test at our institution. Four of them were diagnosed only by cytological sample, with a total of two hundred and twenty-five patients histologically diagnosed. The characteristics of the 225 patients are shown in Table 2. About half of the sampling methods were by EBB/TBB (52.0%), followed by surgical resection (20.9%) and CTNB (15.1%). The majority (70.2%) had adenocarcinoma, and less than half (36.9%) of the patients had clinical stage IVA or IVB or recurrence. The characteristics of the patients whose samples were submitted to each multi-gene panel test are shown in detail in Table A1.

### 3.2. The Submission Rate and the Success Rate of Multi-Gene Panel Tests

**Figure 1:**
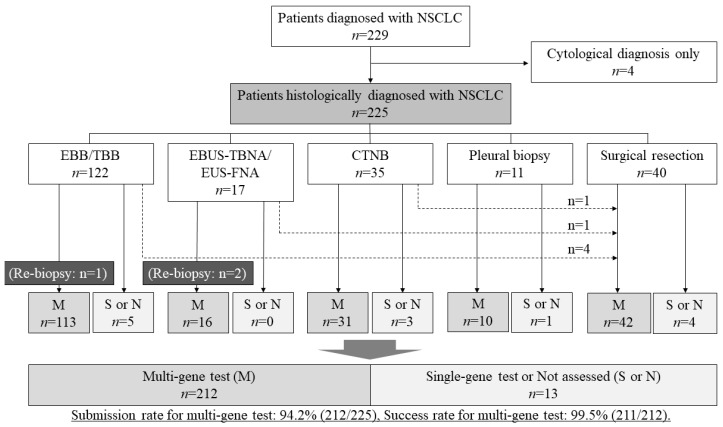
Classification of sampling methods and submitted genetic tests. A total of 225 patients were biopsied by one of five methods: EBB/TBB, EBUS-TBNA/EUS-FNA, CTNB, pleural biopsy, or surgical resection. The submission rate and success rate of the multi-gene panel tests are shown as percentages. The dashed line represents the number of patients that required another biopsy method for submission to the multi-gene panel tests. Abbreviations: NSCLC, non-small-cell lung cancer; EBB, endobronchial biopsy; TBB, transbronchial biopsy; EBUS-TBNA, endobronchial ultrasound-guided transbronchial needle aspiration; EUS-FNA, endoscopic ultrasound-guided fine-needle aspiration; CTNB, computed tomography-guided needle biopsy.

The details of the sampling method for the 225 patients and the submission rate and the success rate of the multi-gene tests are shown in Figure 1. Among the 225 patients, the FFPE samples for 212 patients (94.2%) were submitted to multi-gene panel tests, including 191 samples (84.9%) for the ODxTT and 21 samples (9.3%) for the AmoyDx-multi. Six patients who were histologically diagnosed by small tissue samples in the operable stage received an operation, and their surgical samples were submitted to a multi-gene panel test due to inappropriately small tissue samples. One patient diagnosed by TBB/EBB and two patients diagnosed by EBUS-TBNA in advanced stages required re-biopsy to submit the samples for multi-gene panel tests. The FFPE samples from 13 patients (5.8%) were not submitted for multi-gene panel testing due to an insufficient amount of tumor cells in nine samples, excessive necrosis in two samples, low tumor content in one sample, and the physician’s decision in one sample.

Among the 212 samples submitted for multi-gene panel tests, the overall success rate was 99.5% (211/212). Only one sample obtained by EBUS-TBNA returned invalid RNA results from the ODxTT.

### 3.3. The Results of Multi-Gene Panel Tests

**Figure 2:**
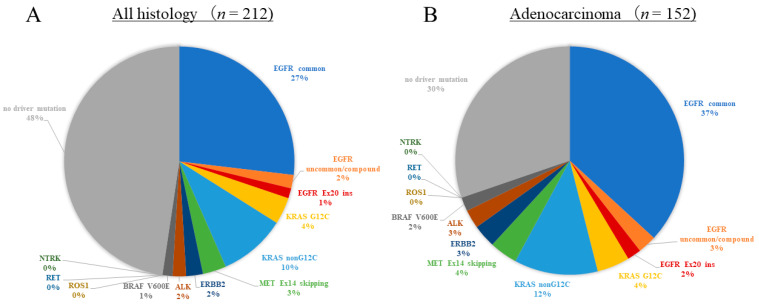
The total detection rates of driver alterations for multi-gene panel tests. (**A**) Detection rate by all histologies. (**B**) Detection rate by adenocarcinoma.

**Figure 3:**
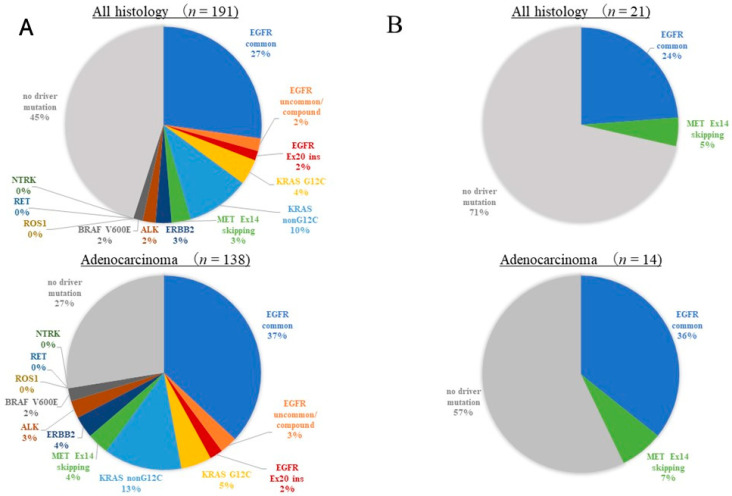
Each detection rate of driver alterations for ODxTT and AmoyDx-multi. (**A**) Detection rate of the ODxTT by all histologies and adenocarcinomas. (**B**) Detection rate of the AmoyDx-multi by all histologies and adenocarcinomas.

The results of the multi-gene panel tests for all histologies and adenocarcinomas are shown in Figure 2. The detection rate of driver oncogene alterations for all histologies was 52.4% (111/212), and that for adenocarcinomas was 69.7% (106/152). Each result of the ODxTT and AmoyDx-multi analyses for all histologies and adenocarcinomas is shown in Figure 3. The detection rate of the ODxTT was 55.0% (105/191), and that of the AmoyDx-multi was 28.6% (6/21) for all histologies. The detection rate was 72.5% (100/138) for the ODxTT and 42.9% (6/14) for the AmoyDx-multi for adenocarcinoma. Among the cases of adenocarcinoma, the details of the detected driver alterations of the ODxTT are as follows: EGFR Ex.19 deletion (*n* = 22, 15.9%), EGFR Ex.21 L858R (*n* = 29, 21.0%), EGFR Ex.20 insertion (*n* = 3, 2.2%), EGFR uncommon/compound mutations (*n* = 4, 2.9%), KRAS G12C mutation (*n* = 7, 5.1%), other KRAS mutations (*n* = 18, 13.0%), MET Ex.14 skipping (*n* = 5, 3.6%), ERBB2 Ex.20 insertion (*n* = 5, 3.6%), ALK fusion (*n* = 4, 2.9%), and BRAF V600E mutation (*n* = 3, 2.2%). The details for the AmoyDx-multi test are as follows: EGFR Ex.19 deletion (*n* = 5, 14.3%), EGFR Ex.21 L858R (*n* = 3, 21.4%), and MET Ex.14 skipping (*n* = 1, 7.1%). In non-adenocarcinoma cases, one EGFR Ex.19 deletion, one KRAS G12C mutation, two KRAS other mutations, and one MET Ex.14 skipping were detected with the ODxTT; meanwhile, no driver alterations were detected with the AmoyDx-multi test.

## 4. Discussion

Compared with an earlier period when the first multi-gene panel test, the ODxTT, was introduced into clinical practice in Japan [9,10,15], our results showed that the submission rate and the success rate of multi-gene panel tests have greatly improved. A recent report suggests that a multi-gene panel test performed prior to the initiation of systemic treatment can potentially enhance prognosis by detecting a wider range of driver oncogene alterations than multiple single-gene tests [5], and therefore the improvement in the submission rate and success rate of multi-gene panel tests is expected to contribute to a good prognosis for patients with NSCLC in clinical practice. This improvement has come from various efforts to submit samples of appropriate quantity and quality [13,16], learning from other reports [12,17], the availability of AmoyDx-multi testing, and improved handling proficiency in laboratory inspections. A previous report showed that tissue size and tumor cell content were significantly associated with a good ODxTT success rate in small biopsy samples, especially when specimens with a tissue size of 4 mm^2^ or larger and a tumor cell content of 20% or more are available [17]. Another report showed that the number of core tissue samples was the most important contributing factor to the success of ODxTT using EBUS-TBNA specimens and recommended that bronchoscopists should perform a sufficient number of punctures to obtain ≥four core tissue samples for successful NGS in EBUS-TBNA [12]. These reports provide important indications for collecting sufficient sample volumes for the ODxTT. For suitable-quality samples, the Japanese Society of Pathology established practical guidelines on the handling of pathological tissue samples for oncologic genome medicine [18]. Furthermore, it has been reported that RNA analysis by ODxTT is more likely to fail for larger specimens, especially for surgical samples [11]. We previously reported that a modified lobectomy sample preparation process could improve the success rate of the ODxTT due to refined RNA quality [13]. Regarding false negative results from multi-gene panel tests related to tumor content, several reports have compared the ODxTT with single-gene tests for EGFR mutations [19,20,21]. The cause of some of the discordant results comes from the difference in the LOD between the tests. As a result, the precise evaluation of tumor content with collaborative work between pathologists and artificial intelligence is expected [22] because recent studies have reported that high variability and low reproducibility exist among individual pathologists [23,24,25]. Furthermore, we also previously reported that some discordant cases between the ODxTT and single-gene testing resulted from the difference in the range of detectable variants between the tests, and these discordant cases had clinically suitable responses to EGFR-TKIs [21]. Real-time PCR in the AmoyDx-multi requires fewer steps than NGS, leading to a shorter TAT, and it is expected that the analysis can be performed with smaller amounts of both DNA and RNA and with a low tumor content. Therefore, the clinical application of the AmoyDx-multi test would broaden the opportunity for patients to obtain the results of multi-gene panel tests. A retrospective study showed that the AmoyDx-multi test had a higher success rate, a shorter turnaround time, and a higher detection rate than NGS panels [26]. Our results showed the detection rate of the AmoyDx-multi test was lower compared with the ODxTT; however, this would depend on the selection criteria for multi-gene panel tests in our institution. In fact, inappropriate specimens might have been submitted for the AmoyDx-multi test in this study, including specimens with an inadequate volume, a low tumor content, and rich necrosis, resulting in false negative results. It is also considered that driver oncogene alterations may be less common in NSCLC with rich necrosis.

Recently, the lung cancer compact panel (LCCP), which is an amplicon-based high-sensitivity next-generation sequencing panel test capable of measuring eight druggable genes, was approved as a companion diagnostic for EGFR mutations, ALK fusions, ROS1 fusions, and MET Ex.14 skipping mutations in November 2022 in Japan. A previous study showed the application of the LCCP for cytological specimens as a prospective validation study [27]. The prospective study revealed a high success rate and favorable detection rate for the test using cytological specimens. The application of cytological specimens will further broaden the opportunity for patients to obtain the results of multi-gene panel testing because there are a certain number of cases in which a sufficient amount of tissue cannot be collected for multi-gene panel tests.

The appropriate timing for performing multi-gene panel tests among patients with early-stage NSCLC is controversial due to problems with the health insurance systems in each country. Ideally, we consider that samples should be submitted for testing at the time of diagnosis. Because it is not always possible to carry out a biopsy from the site of recurrence at the time of recurrence, even if the archived surgical specimens were considered for submission to multi-gene panel tests at the time of recurrence, the nucleic acid in the specimen may have deteriorated over time, especially in early-stage lung cancer, which would take longer to recur. Furthermore, neoadjuvant and perioperative therapies with a combination of immunotherapy and cytotoxic chemotherapy have significantly improved EFS and the complete pathological response compared with conventional neoadjuvant cytotoxic chemotherapy alone, with a manageable safety profile in patients with resectable NSCLC [28,29,30]. Patients with documented EGFR mutations and ALK rearrangements were excluded from these studies. In addition, adjuvant osimertinib therapy among patients with a completed resection of EGFR-mutated NSCLC has already become available in clinical settings as a result of the ADAURA trial [31,32]. Therefore, the driver alteration profile has become information that should be analyzed preoperatively when choosing perioperative therapy. The results of multi-gene panel tests at the time of diagnosis of non-small-cell lung cancer, regardless of stage, will become increasingly important in the future.

There were several limitations to this study. First, this study was a small retrospective study carried out at a single institution; therefore, the generalizability of our results is limited because the specimen sampling strategy and the skill involved in specimen handling will vary in each institution. Further evaluation with a larger multicenter cohort is needed to understand the recent trends in multi-gene panel tests in Japan as a whole. Second, the policy on whether or not to submit samples for multi-gene panel tests varies depending on the stage and histological type between institutions and countries due to problems with the health insurance systems in each country. The results of this study were obtained in the specific medical environment of Japan; therefore, these results may vary in different health care settings or in different situations of accessibility to genetic testing resources and technologies. In our institution, the multi-gene panel tests were performed at the time of diagnosis of NSCLC, regardless of the stage and histological type, for the reasons mentioned above. Third, the accuracy of the results for driver oncogene alterations that are not companion diagnostics for both tests is not guaranteed. Fourth, this study did not analyze the economic costs of genetic testing. Further evaluation of this aspect in Japan would be needed, although a single-center retrospective study in another country reported that NGS is less expensive, more reliable, and requires less tissue than sequential single-gene testing in patients with lung adenocarcinoma [3]. Finally, this study could not compare the performance between the ODxTT and AmoyDx-multi test because the specimens submitted to the AmoyDx-multi test were unfavorable compared with those submitted to the ODxTT.

## 5. Conclusions

The submission rate and success rate of multi-gene panel tests have greatly improved, with a favorable detection rate compared with when the first multi-gene panel test became available in Japan. Future studies are needed to determine the appropriate selection of multi-gene panel tests.

## Figures and Tables

**Table 1:** Submission criteria for multi-gene panel test in our institution.

Tumor Cell Count	Number of Submitted Slides	Selection of Multi-Gene Panel Test
(Cells/Slide)	(8 µm Thickness)	
≤100	Inappropriate for multi-gene testing	
100~200	20 slides	mainly submitted to AmoyDx-multi
200~300	20 slides	mainly submitted to ODxTT (considering AmoyDx-multi in case with low tumor content or rich necrosis)
300~400	15 slides	
400~	10 slides	
Surgical specimens	5–10 slides	

Abbreviations: ODxTT, Oncomine Dx Target Test; AmoyDx-multi, Amoy Dx^®^ Pan Lung Cancer PCR panel.

**Table 2:** Patient characteristics.

Characteristics	Total		Multi-Gene Test		Single-Gene Test or NA	
	*n* = 225	(%)	*n* = 212	(%)	*n* = 13	(%)
Sampling method						
EBB/TBB	117	52.0%	112	52.8%	5	38.5%
Surgical resection	47	20.9%	43	20.3%	4	30.8%
CTNB	34	15.1%	31	14.6%	3	23.1%
EBUS-TBNA/EUS-FNA	16	7.1%	16	7.5%	0	0.0%
Pleural biopsy	11	4.9%	10	4.7%	1	7.7%
Histology						
ADC	158	70.2%	152	71.7%	6	46.2%
Sq	51	22.7%	46	21.7%	5	38.5%
NSCC NOS	16	7.1%	14	6.6%	2	15.4%
Stage (UICC-8)						
0/IA-IB/IIA-IIB/IIIIA-C	137	60.9%	129	60.8%	8	61.5%
Rec/IVA-B	83	36.9%	78	36.8%	5	38.5%
NA	5	2.2%	5	2.4%	0	0.0%

Abbreviations: EBB, endobronchial biopsy; TBB, transbronchial biopsy; CTNB, computed tomography-guided needle biopsy; EBUS-TBNA, endobronchial ultrasound-guided transbronchial needle aspiration; EUS-FNA, endoscopic ultrasound-guided fine-needle aspiration; ADC, adenocarcinoma; Sq, squamous cell carcinoma; NSCC NOS, non-small-cell carcinoma, not otherwise specified; UICC, Union for International Cancer Control; NA, not assessed.

## Data Availability

The data that support the findings of this study are available from the corresponding author upon reasonable request.

## Appendix A

**Table A1:** Patient characteristics for submission to multi-gene panel tests.

Characteristics	Multi-Gene Test		ODxTT		AmoyDx-Multi	
	*n* = 212	(%)	*n* = 191	(%)	*n* = 21	(%)
Sampling method						
EBB/TBB	112	52.8%	103	53.9%	9	42.9%
Surgical resection	43	20.3%	41	21.5%	2	9.5%
CTNB	31	14.6%	24	12.6%	7	33.3%
EBUS-TBNA/EUS-FNA	16	7.5%	14	7.3%	2	9.5%
Pleural biopsy	10	4.7%	9	4.7%	1	4.8%
Histology						
ADC	152	71.7%	138	72.3%	14	66.7%
Sq	46	21.7%	43	22.5%	3	14.3%
NSCC NOS	14	6.6%	10	5.2%	4	19.0%
Stage (UICC-8)						
0/IA-IB/IIA-IIB/IIIIA-C	129	60.8%	116	60.7%	13	61.9%
Rec/IVA-B	78	36.8%	71	37.2%	7	33.3%
NA	5	2.4%	4	2.1%	1	4.8%

Abbreviations: EBB, endobronchial biopsy; TBB, transbronchial biopsy; CTNB, computed tomography-guided needle biopsy; EBUS-TBNA, endobronchial ultrasound-guided transbronchial needle aspiration; EUS-FNA, endoscopic ultrasound-guided fine-needle aspiration; ADC, adenocarcinoma; Sq, squamous cell carcinoma; NSCC NOS, non-small-cell carcinoma, not otherwise specified; UICC, Union for International Cancer Control; NA, not assessed.

## References

[1] Kris M.G., Johnson B.E., Berry L.D., Kwiatkowski D.J., Iafrate A.J., Wistuba I.I., Varella-Garcia M., Franklin W.A., Aronson S.L., Su P.-F. (2014). Using multiplexed assays of oncogenic drivers in lung cancers to select targeted drugs. JAMA.

[2] Takeda M., Sakai K., Terashima M., Kaneda H., Hayashi H., Tanaka K., Okamoto K., Takahama T., Yoshida T., Iwasa T. (2015). Clinical application of amplicon-based next-generation sequencing to therapeutic decision making in lung cancer. Ann. Oncol..

[3] Dall’Olio F.G., Conci N., Rossi G., Fiorentino M., De Giglio A., Grilli G., Altimari A., Gruppioni E., Filippini D.M., Di Federico A. (2020). Comparison of Sequential Testing and Next Generation Sequencing in advanced Lung Adenocarcinoma patients—A single centre experience. Lung Cancer.

[4] Yu T.M., Morrison C., Gold E.J., Tradonsky A., Layton A.J. (2018). Multiple biomarker testing tissue consumption and completion rates with single-gene tests and investigational use of Oncomine Dx target test for advanced non-small-cell lung cancer: A single-center analysis. Clin. Lung Cancer.

[5] Kanasaki H., Ozawa Y., Nakamura N., Nagasaki K., Matsuyama W., Akahori D., Niwa M., Ogasawara T., Sato J. (2024). Upfront Multiplex Gene Test Helps Prolong Survival in Advanced Non-small Cell Lung Cancer. Anticancer Res..

[6] Yatabe Y., Sunami K., Goto K., Nishio K., Aragane N., Ikeda S., Inoue A., Kinoshita I., Kimura H., Sakamoto T. (2020). Multiplex gene-panel testing for lung cancer patients. Pathol. Int..

[7] Murakami S., Yokose T., Nemoto D., Suzuki M., Usui R., Nakahara Y., Kondo T., Kato T., Saito H. (2021). Suitability of bronchoscopic biopsy tissue samples for next-generation sequencing. Diagnostics.

[8] Kunimasa K., Matsumoto S., Nishino K., Nakamura H., Kuhara H., Tamiya M., Inoue T., Kawamura T., Kawachi H., Kuno K. (2020). Improvement strategies for successful next-generation sequencing analysis of lung cancer. Future Oncol..

[9] Sakata S., Otsubo K., Yoshida H., Ito K., Nakamura A., Teraoka S., Matsumoto N., Shiraishi Y., Haratani K., Tamiya M. (2022). Real-world data on NGS using the Oncomine DxTT for detecting genetic alterations in non-small-cell lung cancer: WJOG13019L. Cancer Sci..

[10] Takahashi T., Nishio M., Nishino K., Yoshiki Y., Shiraiwa N., Emir B., Iadeluca L., Yatabe Y., Nishio K. (2023). Real-world study of next-generation sequencing diagnostic biomarker testing for patients with lung cancer in Japan. Cancer Sci..

[11] Hatanaka Y., Kinoshita I., Amemiya K., Dosaka-Akita H. (2022). Predictive Biomarker Testing for Lung Cancer: Past and Future Perspectives. JJLC.

[12] Uchimura K., Yanase K., Imabayashi T., Takeyasu Y., Furuse H., Tanaka M., Matsumoto Y., Sasada S., Tsuchida T. (2021). The Impact of Core Tissues on Successful Next-Generation Sequencing Analysis of Specimens Obtained through Endobronchial Ultrasound-Guided Transbronchial Needle Aspiration. Cancers.

[13] Sakaguchi T., Iketani A., Furuhashi K., Nakamura Y., Suzuki Y., Ito K., Fujiwara K., Nishii Y., Katsuta K., Taguchi O. (2021). A method to improve genetic analysis of lung cancer samples. Respirology.

[14] Food and Drug Administration (2017). Summary of Safety and Effectiveness Data. https://www.accessdata.fda.gov/cdrh_docs/pdf16/P160045B.pdf.

[15] Sakamoto T., Matsubara T., Takahama T., Yokoyama T., Nakamura A., Tokito T., Okamoto T., Akamatsu H., Oki M., Sato Y. (2023). Biomarker testing in patients with unresectable advanced or recurrent non-small cell lung cancer. JAMA Netw. Open.

[16] Sakaguchi T., Nishii Y., Iketani A., Esumi S., Esumi M., Furuhashi K., Nakamura Y., Suzuki Y., Ito K., Fujiwara K. (2022). Comparison of the analytical performance of the Oncomine dx target test focusing on bronchoscopic biopsy forceps size in non-small cell lung cancer. Thorac. Cancer.

[17] Takeyasu Y., Yoshida T., Motoi N., Teishikata T., Tanaka M., Matsumoto Y., Shinno Y., Okuma Y., Goto Y., Horinouchi H. (2021). Feasibility of next-generation sequencing (Oncomine™ DX Target Test) for the screening of oncogenic mutations in advanced non-small-cell lung cancer patients. Jpn. J. Clin. Oncol..

[18] Hatanaka Y., Kuwata T., Morii E., Kanai Y., Ichikawa H., Kubo T., Hatanaka K.C., Sakai K., Nishio K., Fujii S. (2021). The Japanese Society of Pathology Practical Guidelines on the handling of pathological tissue samples for cancer genomic medicine. Pathol. Int..

[19] Murakami S., Yokose T., Shinada K., Isaka T., Katakura K., Ushio R., Kondo T., Kato T., Ito H., Saito H. (2022). Comparison of next-generation sequencing and cobas EGFR mutation test v2 in detecting EGFR mutations. Thorac. Cancer.

[20] Kanaoka K., Tamiya A., Inagaki Y., Taniguchi Y., Nakao K., Takeda M., Matsuda Y., Okishio K., Shimizu S. (2023). Possible False Results with cobas^®^ EGFR Mutation Test v2 and Oncomine Dx Target Test for EGFR Mutation. Anticancer Res..

[21] Sakaguchi T., Iketani A., Esumi S., Esumi M., Suzuki Y., Ito K., Fujiwara K., Nishii Y., Katsuta K., Yasui H. (2023). Clinical importance of the range of detectable variants between the Oncomine Dx target test and a conventional single-gene test for EGFR mutation. Sci. Rep..

[22] Sakamoto T., Furukawa T., Pham H.H.N., Kuroda K., Tabata K., Kashima Y., Okoshi E.N., Morimoto S., Bychkov A., Fukuoka J. (2022). A collaborative workflow between pathologists and deep learning for the evaluation of tumour cellularity in lung adenocarcinoma. Histopathology.

[23] Mikubo M., Seto K., Kitamura A., Nakaguro M., Hattori Y., Maeda N., Miyazaki T., Watanabe K., Murakami H., Tsukamoto T. (2020). Calculating the Tumor Nuclei Content for Comprehensive Cancer Panel Testing. J. Thorac. Oncol..

[24] Smits A.J., Kummer J.A., de Bruin P.C., Bol M., van den Tweel J.G., Seldenrijk K.A., Willems S.M., Offerhaus G.J., de Weger R.A., van Diest P.J. (2014). The estimation of tumor cell percentage for molecular testing by pathologists is not accurate. Mod. Pathol..

[25] Viray H., Li K., Long T.A., Vasalos P., Bridge J.A., Jennings L.J., Halling K.C., Hameed M., Rimm D.L. (2013). A prospective, multi-institutional diagnostic trial to determine pathologist accuracy in estimation of percentage of malignant cells. Arch. Pathol. Lab. Med..

[26] Kunimasa K., Matsumoto S., Kawamura T., Inoue T., Tamiya M., Kanzaki R., Maniwa T., Okami J., Honma K., Goto K. (2023). Clinical application of the AMOY 9-in-1 panel to lung cancer patients. Lung Cancer.

[27] Morikawa K., Kida H., Handa H., Inoue T., Saji H., Koike J., Nakamura S., Sato Y., Ueda Y., Suzuki F. (2022). A Prospective Validation Study of Lung Cancer Gene Panel Testing Using Cytological Specimens. Cancers.

[28] Forde P.M., Spicer J., Lu S., Provencio M., Mitsudomi T., Awad M.M., Felip E., Broderick S.R., Brahmer J.R., Swanson S.J. (2022). Neoadjuvant Nivolumab plus Chemotherapy in Resectable Lung Cancer. N. Engl. J. Med..

[29] Provencio M., Nadal E., González-Larriba J.L., Martínez-Martí A., Bernabé R., Bosch-Barrera J., Casal-Rubio J., Calvo V., Insa A., Ponce S. (2023). Perioperative Nivolumab and Chemotherapy in Stage III Non-Small-Cell Lung Cancer. N. Engl. J. Med..

[30] Wakelee H., Liberman M., Kato T., Tsuboi M., Lee S.H., Gao S., Chen K.N., Dooms C., Majem M., Eigendorff E. (2023). Perioperative Pembrolizumab for Early-Stage Non-Small-Cell Lung Cancer. N. Engl. J. Med..

[31] Wu Y.L., Tsuboi M., He J., John T., Grohe C., Majem M., Goldman J.W., Laktionov K., Kim S.W., Kato T. (2020). Osimertinib in Resected EGFR-Mutated Non-Small-Cell Lung Cancer. N. Engl. J. Med..

[32] Tsuboi M., Herbst R.S., John T., Kato T., Majem M., Grohé C., Wang J., Goldman J.W., Lu S., Su W.C. (2023). Overall Survival with Osimertinib in Resected EGFR-Mutated NSCLC. N. Engl. J. Med..

